# *PKD1* 5’UTR variants are a rare cause of disease in ADPKD and suggest a new focus for therapeutic development

**DOI:** 10.1038/s41431-025-01949-z

**Published:** 2025-09-26

**Authors:** Laura Wedd, Yvonne Hort, Chirag Patel, John A. Sayer, Rocio Rius, Andrew J. Mallett, Denny L. Cottle, Ian M. Smyth, Timothy Furlong, John Shine, Amali Mallawaarachchi

**Affiliations:** 1 Molecular Genetics of Inherited Kidney Disorders Laboratory, Garvan Institute of Medical Research, Sydney, NSW Australia; 2 Centre for Population Genomics, Garvan Institute of Medical Research and UNSW Sydney, Sydney, NSW Australia; 3 Centre for Population Genomics, Murdoch Children’s Research Institute, Melbourne, VIC Australia; 4 Genetic Health Queensland, Royal Brisbane and Women’s Hospital, Brisbane, QLD Australia; 5 Biosciences Institute, Newcastle University, Newcastle upon Tyne, UK; 6 Renal services, The Newcastle upon Tyne Hospitals NHS Foundation Trust, Newcastle, UK; 7 NIHR Bioresource Centre, Newcastle upon Tyne, UK; 8 Department of Renal Medicine, Townsville University Hospital, Townsville, QLD Australia; 9 College of Medicine and Dentistry, James Cook University, Townsville, QLD Australia; 10 Institute for Molecular Bioscience, The University of Queensland, Brisbane, QLD Australia; 11 Development and Stem Cells Program, Monash Biomedicine Discovery Institute & Department of Anatomy and Developmental Biology, Monash University, Clayton, VIC Australia; 12 Department of Clinical Genetics, Royal Prince Alfred Hospital, Sydney, NSW Australia; 13 School of Clinical Medicine, UNSW Medicine & Health, UNSW Sydney, Sydney, NSW Australia; 14 Sydney Medical School, Faculty of Medicine and Health, University of Sydney, Sydney, NSW Australia

**Keywords:** Gene regulation, Medical genomics, Genetic testing

## Abstract

Autosomal Dominant Polycystic Kidney Disease (ADPKD), caused by pathogenic variants in *PKD1* and *PKD2*, is the most common monogenic cause of kidney failure. Approximately 10% of ADPKD patients remain undiagnosed after coding-region focused genomic testing. Non-coding variants in regulatory regions are not an established cause of disease in ADPKD. We performed regulatory region analysis in a primary cohort of undiagnosed ADPKD patients (*n* = 20) and then extended this analysis to patients with undiagnosed cystic kidney disease within the Australian KidGen cohort (*n* = 42) and the Genomics England rare disease cohort (*n* = 1320). Through this genomic analysis we identified two rare, potentially disease-causing variants in the *PKD1* 5′untranslated region (UTR). We then designed a PKD1 5′UTR-luciferase translation assay to characterise these variants in vitro, which showed that a *PKD1* variant c.−69dupG, reduced the translation efficiency of the main PKD1 open reading frame by ~87% compared to wildtype (*p* < 0.0001). The human *PKD1* 5′UTR contains two upstream open reading frames (uORFs). Using our model, we knocked-out the upstream open reading frames of the wildtype *PKD1* 5′UTR sequence, which increased expression of wildtype polycystin-1 (130%, *p* < 0.0001). We show that *PKD1* 5′-UTR variants are a currently overlooked rare cause of disease in ADPKD and that analysis of this region should be included in variant analysis pathways to increase diagnostic rates. In addition, we show that manipulation of the wildtype 5′UTR sequence can increase polycystin-1 expression, providing insights into regulation of *PKD1* and suggested new approaches for therapeutic intervention in this haplo-insufficient disease.

## Introduction

It is estimated that between 1 in 500–2500 people are clinically affected by autosomal dominant polycystic kidney disease (ADPKD, MONDO:0004691) [1]. Heterozygous, pathogenic loss-of-function variants in either *PKD1* or *PKD2* are the primary cause of typical ADPKD, which is characterised by development of progressive macroscopic kidney cysts that cause massively enlarged kidneys and kidney failure [2].

Approximately ten percent of individuals who undergo standard clinical genetic testing for ADPKD will remain without a genetic diagnosis [3]. This is in part due to the technical limitations of current diagnostic testing, which most commonly utilises short-read whole exome or capture-based sequencing methods, with a focus on coding regions of known genes [3]. Increasingly, it has become apparent across all rare disease cohorts that challenges associated with identifying and interpreting non-coding variants contribute to the lack of a genetic diagnosis [4]. The application of whole genome sequencing and robust analysis and functional characterisation of non-coding regions of *PKD1* has been shown to identify pathogenic atypical splicing variants, that were a previously unidentified cause of disease [3]. Non-coding variants outside of those predicted to impact splicing, such as regulatory regions and promoter variants, are currently overlooked as a potential cause of disease in ADPKD.

The 5′untranslated region (5′UTR) of protein coding genes has long been recognised as critical for regulating translation [5, 6]. In eukaryotes the ribosomal preinitiation complex scans across the 5′UTR and initiates translation upon encountering an AUG start codon. Whether or not translation initiates from any given AUG, whilst influenced by multiple factors, is particularly dependent on the surrounding sequence context, especially the secondary structure and Kozak consensus sequence around the AUG [5, 7].

Approximately half of all 5’UTRs contain short open reading frames (ORF), known as upstream open reading frames (uORFs) that are potentially translated, but terminate before the start codon of the coding sequence (CDS) or main ORF. uORFs regulate the translation of the main ORF in a complex and context-dependent manner [8–10] and predominately act in an inhibitory fashion. In many genes studied, translation of uORFs has been found to reduce protein expression levels by between 30 and 80% [11–13].

Genomic variants which modify or create uORFs within the 5′UTR can cause human disease. The creation of AUG start sites introducing a novel uORF that inhibits translation of the main ORF has been demonstrated to cause disease in several genetic conditions including Cornelia De Lange, Saethre-Chotzen syndrome and *MEF2C*-associated developmental disorder [14–17]. Similarly, variants which disrupt existing uORFs by removing an in-frame stop codon can cause read-through and repression of translation from the main ORF and have a loss-of-function effect (i.e. result in loss or major reduction of the amount of wildtype protein) [16, 18, 19]. More rarely, uORFs encoding functional peptides may be disrupted and have gain-of-function effects - for example, variants in an uORF of a gene that result in the loss of the wildtype suppressive effect of that uORF, resulting in increase in gene expression [20, 21].

In addition to understanding new pathogenic mutational mechanisms, investigation of regulatory regions offers an opportunity to better understand mechanisms that control gene expression. In other disease groups, investigation of regulatory regions, such as the 5′UTR, has also led to opportunities for therapeutic developments. For example, in disease models of both cystic fibrosis and cardiac hypertrophy, antisense oligonucleotides have been used to target the 5′UTR uORFs in the relevant genes and this therapy has been able to increase wildtype protein expression [22–24].

In this study, we identified a potential uORF disrupting variant in the *PKD1* gene of an undiagnosed ADPKD patient. We developed an in vitro assay supporting that this variant significantly reduces PKD1 expression. This is the first example of a pathogenic 5′UTR variant in ADPKD, showing that these variants are a currently overlooked cause of disease. To further characterise this finding, we designed novel sequence alterations to rescue the effect of the pathogenic variant in our cell culture model. In addition to being able to rescue the impact of the patient variant, we demonstrate through our model that alteration of the wildtype *PKD1* 5’UTR sequence can increase expression of wildtype polycystin-1, providing insights into regulation of *PKD1* and opening the door for harnessing this for therapeutic intervention in this haplo-insufficient disease [25].

## Materials and methods

### Characterisation of the wildtype PKD1 5′ UTR

The *PKD1* MANE select transcript (NM_001009944.3) was used to define the *PKD1* 5′UTR as chr16 (GRCh38):2,135,690–2,135,898. To identify potential existing uORFs within the 5′UTR, short ORFs with an AUG start codon and in-frame stop codon were assessed. Alternative start sites such as CUG, were not considered. The *Catalog of predicted functional upstream open reading frames in humans* [26] which integrates ribosomal profiling and mass spectrometry data to identify functional uORFs, was interrogated to assess whether the *PKD1* uORFs had evidence for functionality. To assess the degree of sequence conservation of uORFs, we extracted basewise PhyloP and elementwise PhastCons scores from the UCSC 470-mammal multiple sequence alignments (‘phyloP470way’ and ‘phastCons470way’ tracks) [27]. Consistent with prior analyses of uORF conservation and reflecting the predominantly mammalian conservation of uORFs, we restricted our analysis to the mammalian subset of the alignment [28, 29]. Conservation thresholds were set at PhyloP >2.0, indicating purifying selection, and PhastCons >0.8, indicating conserved elements [29, 30].

Kozak sequence strength was scored by matching the –3 and +4 positions around the AUG to the core motif A/GXXATGG [5, 7]. Contexts are classified as strong if both the –3 and +4 positions match the consensus, moderate if only one does, and weak if neither does [31]. Mammalian uORF peptides were aligned using Jalview (v2.11.4.1) and mean percent identity calculated to classify conservation as high (≥80%), moderate (50–80%), or weak (<50%) [32].

### Recruitment and genomic sequencing of undiagnosed ADPKD patients

Genomic analysis was undertaken in an undiagnosed ADPKD cohort within the Garvan Institute of Medical Research, Sydney, Australia (Garvan). Short-read whole genome sequencing was performed for all (see Supplementary Methods) [33, 34]. Patients with a typical ADPKD phenotype, with or without a significant family history, were recruited after being undiagnosed following clinical sequencing of *PKD1* and *PKD2*. After informed consent, they were recruited for research investigation, which included research variant analysis.

To identify *PKD1* 5′UTR variants in other undiagnosed ADPKD cohorts, 5′UTR analysis was undertaken in undiagnosed cystic-phenotype patients within the KidGen and Genomics England rare disease cohorts [35, 36]. The KidGen cohort consisted of 42 undiagnosed Australian patients with a cystic kidney disease phenotype. Those with a clinical diagnosis possibly consistent with *PKD1-*associated ADPKD were identified by assessing available medical records. Variant analysis was restricted to the *PKD1* 5′UTR utilising available WGS or WES data. The Genomics England rare disease cohort consisted of 1320 cystic disease patients who had already undergone diagnostic genomic analysis. Variant analysis of this cohort was restricted to the *PKD1* 5′UTR utilising available WGS data.

Ethics approval for the Garvan cohort was obtained from RPAH Human Research Ethics Committee (HREC/18/RPAH/726) and for the KidGen Cohort from the Royal Children’s Hospital and Melbourne Health Human Research Ethics Committees (HREC/83945/RCHM-2022; HREC/16/MH/251). All participants in the Genomics England rare disease cohort provided written consent to access their anonymized clinical and genomic data for research purposes. The project model and its informed consent process have been approved by the National Research Ethics Service, Research Ethics Committee for East of England (Cambridge South Research Ethics Committee).

### Variant analysis

Within the Garvan cohort, Initial variant analysis was targeted to coding and non-coding regions of *PKD1* (NM_001009944.3) and *PKD2* (NM_000297.4); PKD-related genes associated with atypical disease were also analysed (including *ALG5, ALG8, ALG9, COL4A1, DNAJB11, DZIP1L, GANAB, HNF1B, IFT140, NEK8, PKHD1, PMM2, PRKCSH, SEC63, TSC1, TSC2, UMOD, VHL*). Rare variants (allele frequency <0.001, in gnomAD v4.1) were manually reviewed and prioritised according to American College of Medical Genetics (ACMG) Guidelines for pathogenicity [37]. SpliceAI or Introme were used to assess variants predicted to impact splicing [38, 39].

### 5′UTR variant analysis

Analysis of the *PKD1* 5′UTR was performed in the Garvan, KidGen and Genomics England 100,000 Genomes cohorts. Rare variants (allele frequency <0.001, in gnomAD v4.1) identified within the *PKD1* 5′UTR were reviewed by a variant analyst. VuTR was used to visualise the impact of identified rare variants [19, 40]. AUG-creating variants within the 5′UTR or variants predicted to disrupt the stop codon of existing uORFs were prioritised as ‘potentially disease-causing’. Variants which met any of the following criteria were flagged as ‘of uncertain significance’: (1) AUG-creating variants which cause an N-terminal extension, (2) variants which result in an amino acid change within a defined uORF (since amino acid substitutions within uORFs have been reported to alter the dynamics of ribosome stalling [41] and have been associated with human disease previously [20]), (3) variants altering the Kozak sequence motif of a defined uORF or the main ORF (because an alteration to translational efficiency cannot be completely excluded), (4) start-loss variants of a defined uORF, and (5) any other rare variants (allele frequence <0.001) within the *PKD1* 5’UTR.

### PKD1 5′UTR variation in publicly available datasets

ClinVar and the PKD Disease Database were interrogated for rare variants (Allele frequency <0.001, access date 31/01/2024) within the *PKD1* 5′UTR region that were predicted to disrupt or create a uORF. The gnomAD dataset (v4.0, access date 31/01/2024 [42]) was also interrogated to assess for the possibility of a potentially disease-causing variant within the *PKD1* 5′UTR region. In order to define ‘rare’ variants within the gnomAD dataset, the tolerated allele frequency for variation in gnomAD v.4.0 was assessed utilising https://www.cardiodb.org/allelefrequencyapp/ considering a prevalence of 1 in 1000, allelic heterogeneity 0.1 and genetic heterogeneity 0.9 [43]. Whilst *PKD1-*associated ADPKD is considered completely penetrant by adulthood, a penetrance of 0.75 was utilised to account for varied age distribution and the lack of phenotype data associated with population databases. 5’UTR variant analysis was then performed for gnomADv4.0 variants of an allele frequency of <6 × 10^−5^. We subsequently assessed all gnomAD v4.1 variation (access date 21/06/2024) within the *PKD1* defined uORFs to exclude the possibility that a common genomic variation might occur.

### Expression constructs

A *PKD1* 5′UTR luciferase translation assay was used to characterize uncertain or suspected pathogenic variants identified through the manual curation process across the *PKD1* 5′UTR (See Supplementary Table 1).

Expression constructs were designed by introducing candidate patient variants to the *PKD1* 5′UTR wildtype sequence. For variants that appeared to be pathogenic by this approach, additional validation of variant pathogenicity was performed by introducing variants predicted to ‘rescue’ the effect of the original patient variant and measuring this effect. All candidate variants assessed were compared to wildtype, and a predicted benign variant (c.−52C>T). This variant was predicted benign based on benign in silico predictions, a high allele frequency in gnomAD and high frequency of homozygotes in gnomAD (Supplementary Table 1). To further characterise the native function of the *PKD1* 5′ UTR, constructs were designed with variants introduced to alter the wildtype 5′UTR uORFs.

The 5′UTR variants were cloned directly upstream of Gaussia luciferase (GLuc) in the modified pEZX-GA02 plasmid (Genecopoeia; sequence can be supplied on request) and sequenced. Secreted alkaline phosophatase (SeAP) is expressed on the same construct for normalisation of the transfection efficiency.

### Cell culture, transfection and analysis

Luciferase constructs were transfected into HEK 293T/17 (CRL-11268 from ATCC, grown in Dulbecco’s Modified Eagle Medium, High Glucose plus pyruvate, with 10% foetal bovine serum) using Lipofectamine 3000, following the manufacturer’s directions. Cells were transfected at ~80% confluence for 24 h. The transfection media was changed, and 24-h media collected and assayed for Gluc and SeAP simultaneously using the Secrete-Pair Dual Luminescence Assay (Genecopoeia). 12 replicates of each variant were transfected, in two independent experiments, and duplicates of each transfection assayed. The wildtype variant was used as a control with each transfection. Statistical analysis was performed utilizing a 1-way ANOVA followed by Tukey’s HSD test using GraphPad Prism 10.

## Results

### Characterisation of the PKD1 5′UTR

**Fig. 1:**
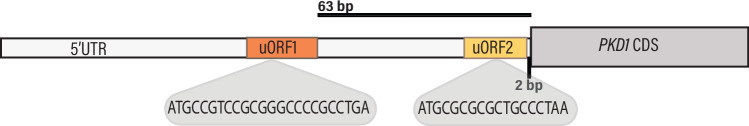
*PKD1* 5'UTR and putative uORFs. Schematic showing the *PKD1* 5′UTR and putative uORFs, uORF1 (orange) and uORF2 (yellow). The uORF sequence is shown in the grey triangles. Black lines show distance at which each uORF terminates from the main ORF (63 bp for uORF1, and 2 bp for uORF2). CDS coding sequence.

Prior to performing variant analysis, we utilized available reference data to characterize key aspects of the wildtype *PKD1* 5′UTR region. Two putative uORFs within the 5′UTR of *PKD1* were identified. uORF1 is located at chr16(GRCh38):2,135,753–2,135,776 encoding a 7 amino acid peptide MPSAGPA[stop] in-frame with the main ORF, terminating 63 base pairs (bp) upstream of the main ORF AUG (mAUG), with uAUG1 122 bp from the 5′cap. uORF2 is located at chr16(GRCh38):2,135,692–2,135,709 encoding a 5 amino acid peptide MRALP[stop], out-of-frame with the main ORF, terminating 2 bp upstream of the mAUG, with uAUG2 189 bp from the 5′cap (Fig. 1). Both uORF1 and uORF2 have evidence supporting functionality [26]. Strong species conservation (PhyloP score >0.6) was observed for the AUG and stop codons of both uORFs. Kozak strength of both uORF1 and uORF2 was scored as moderate as they both have G at –3 and C at +4 positions around the AUG to the core motif A/GXXATGG as visualized in VuTR [40]. The predicted peptide sequence for both uORFs demonstrated moderate (>70%) conservation in mammalian alignment analysis.

### Identification and investigation of rare PKD1 5′UTR variants in ADPKD cohorts

**Fig. 2:**
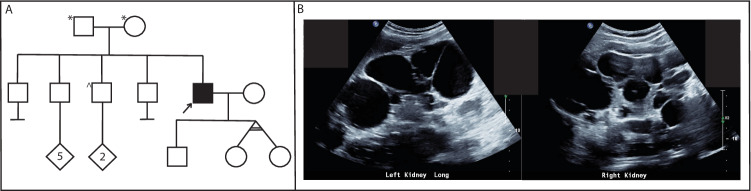
Pedigree of RBW402. **A** Pedigree of RBW402, clinically diagnosed with ADPKD. No significant family history reported with parents having had normal ultrasound examinations in their 60 s. *normal ultrasound examination in 60’s. ^ normal ultrasound examination in 30’s. Arrow indicates proband (RBW402). **B** Kidney ultrasound images from RBW402 demonstrating bilateral kidney cysts.

We identified via genome sequencing analysis a single nucleotide duplication within the *PKD1* 5′UTR, NM_001009944.3:c.−69dupG; NC_000016.10:g.2135757_2135758insC (Dec.2013: hg38, GRCh38) in a male proband (RBW402, supplementary Fig. 1) with a clinical diagnosis of ADPKD, without a significant family history (Fig. 2A). His parents had normal ultrasound examinations in their 60’s, but parental samples were not available to confirm whether this variant occurred de novo. His clinical history is consistent with typical ADPKD; he was diagnosed at 39 years old with >20 bilateral kidney cysts causing enlarged kidney lengths (19 cm left kidney length and 22 cm right kidney length), with liver cysts (Fig. 2B). Clinical ADPKD panel testing via short-read WGS (*PKD1, PKD2, GANAB, HNF1B, TSC1, TSC, OFD1, UMOD, PKHD1)*, and targeted sequencing and MLPA of *HNF1B* did not identify a genetic cause of his ADPKD.

**Fig. 3:**
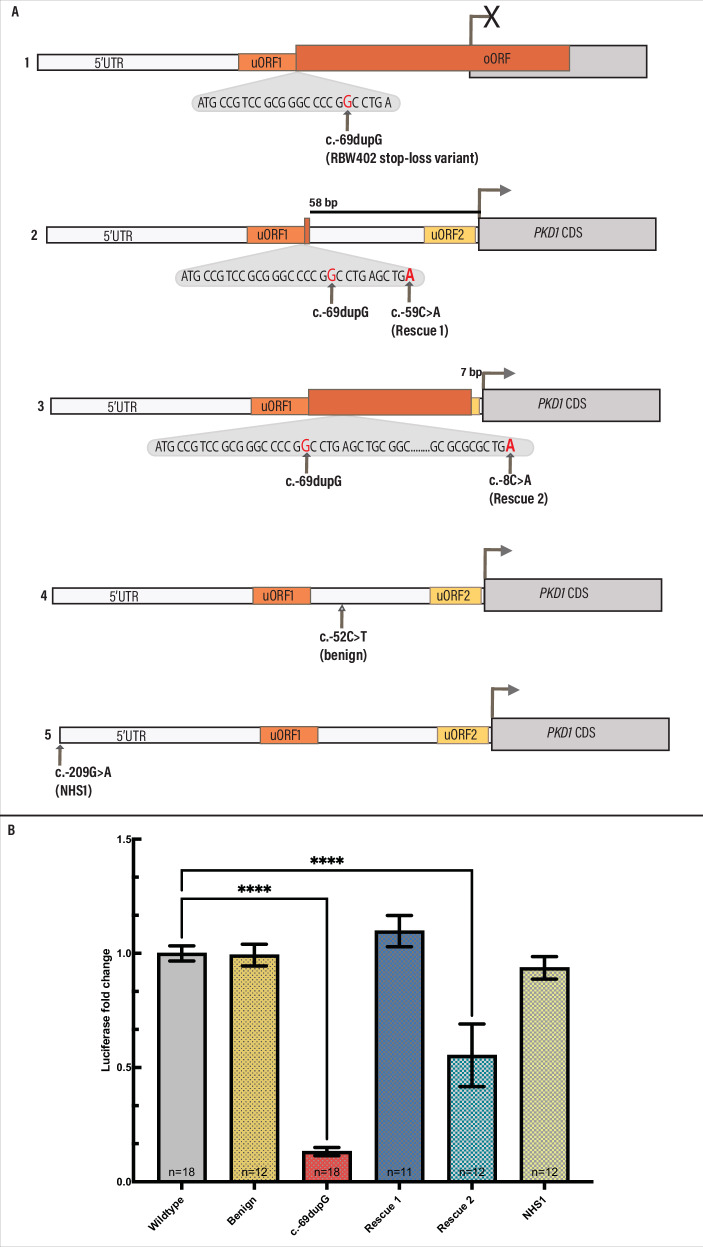
*PKD1* 5'UTR variants modelled. **A** Schematic showing the predicted impact of variants modelled in the luciferase reporter assay. (1) RBW402 variant c.−69dupG predicted to create an overlapping open reading frame (oORF) which reads through the *PKD1* main ORF. (2) Rescue 1, c.−59C>A in phase with the c.−69dupG variant, predicted to cause an extension of uORF1, terminating 58 bp before the main ORF. 3) Rescue 2, c.−8C>A in phase with the c.−69dupG variant, predicted to cause an extension of uORF1, terminating 7 bp before the main ORF. 4) c.−52C>T (Benign) and 5) c.−209G>A (NHS1) not predicted to disrupt *PKD1* uORFs. uORF upstream open reading frame; CDS Coding sequence. **B** Luciferase activity of modelled variants relative to wildtype. Bars represent mean with standard error of the mean. RBW402 patient variant c.−69dupG was found to significantly reduce luciferase activity relative to wildtype. Rescue 1 recovered activity and rescue 2 partially rescued activity. No significant difference was observed between the NHS1 or benign variants and wildtype. **** *p* < 0.0001.

The c.−69dupG variant is absent from gnomAD v4.1 and is not predicted to impact splicing (SpliceAI <0.05). Comparison of the translated uORF1 sequences using the Expasy Tool found that this variant introduces a frameshift within uORF1, causing a stop-loss and is predicted to cause read-through of the main ORF, out-of-frame (Fig. 3, Supplementary Table 1) [44]. No rare coding variants in *PKD1* or *PKD2* were identified. A rare *PKD2* intronic variant and *PKD1* intronic variant were also identified, but neither were predicted to impact splicing (Supplementary Table 2). The c.−69dupG variant was confirmed by Sanger sequencing (data not shown).

Another rare 5′UTR variant, c.−209G>A, was identified in an individual with bilateral, multicystic, enlarged kidneys and a clinical diagnosis of ADPKD within the Genomics England rare disease cohort (NHS1). This variant was interpreted as of uncertain significance based on in silico analyses (Fig. 3A and Supplementary Table 1).

No other predicted uORF-disrupting or uORF-creating variants were identified in the KidGen or Garvan undiagnosed ADPKD cohorts, in ClinVar or the PKD variant database (Supplementary Table 3). Further, we did not identify any predicted uORF-disrupting variants in the gnomAD v4.1 database that were predicted to cause read-through of the main ORF (Supplementary Table 4), nor did we identify any rare putative AUG-creating variants in the gnomAD v4.0 dataset (Supplementary Table 5 and Supplementary results, Section 1).

### In vitro assessment of identified PKD1 5′UTR variants

To characterize the predicted uORF-disrupting c.−69dupG variant identified in patient RBW402, we designed a construct to mimic the patient variant, which was cloned upstream of a luciferase reporter gene (Fig. 3A). We compared the luciferase activity of the patient variant (c.−69dupG) to wildtype and a predicted benign variant (c.−52C>T, Fig. 3A). The c.−52C>T variant was selected as a negative control, benign variant based on benign in silico predictions and high allele frequency in gnomAD. We found that the patient variant (c.−69dupG) significantly reduced the translation efficiency of the main ORF by ~87% when compared to wildtype (mean difference 0.8678, *p* < 0.0001, by Tukey HSD test, Fig. 3B). There was no statistically significant difference in luciferase activity between wildtype and the c.−52C>T variant selected as a negative control (mean difference 0.008, *p* > 0.99, by Tukey HSD test, Fig. 3B).

To further validate that the variant identified in RBW402 caused a significant reduction in polycystin-1 translation, we then assessed the c.−69dupG variant in-phase with two different variants designed to rescue the effect of the predicted pathogenic patient variant. Rescue of the patient c.−69dupG variant through the introduction of an in-frame stop codon ending 58 nucleotides upstream of the translation start site of the main ORF, ‘Rescue 1’, recovered luciferase expression, with a minor increase in luciferase activity observed between Rescue 1 and wildtype (mean difference −0.097, *p* = 0.0034, by Tukey HSD test). The introduction of a stop codon ending 7 nucleotides upstream of the translation start site, ‘Rescue 2’, only partially rescued activity, with ~45% reduction in activity observed (mean difference 0.4465, *p* < 0.0001, by Tukey HSD test). These data are consistent with the c.−69dupG disrupting translation of the main ORF via read-through.

We modelled the c.−209G>A variant identified in patient NHS1 in the luciferase reporter assay and did not find a significant difference between the NHS1 variant and wildtype, supporting that this variant is likely benign (mean difference 0.06369, *p* = 0.1315, by Tukey HSD test) (Fig. 3B).

### Manipulation of wildtype 5′UTR sequence increases polycystin-1 expression

**Fig. 4:**
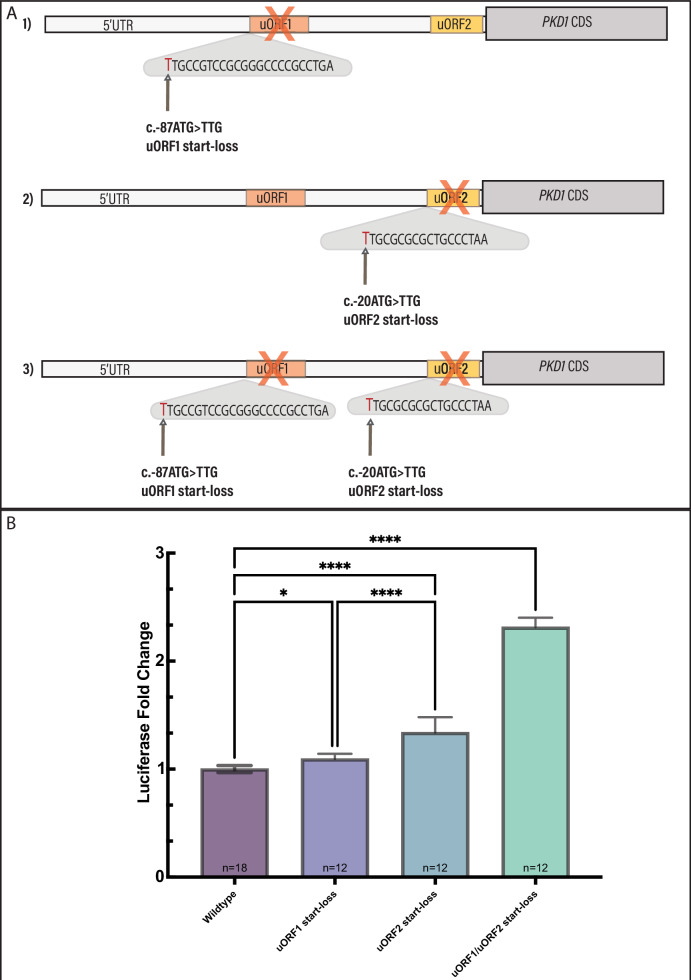
Impact of uORF knock-out (start-loss) variants. **A** Schematic of constructs designed to knock-out uORF1 (1), uORF2 (2) and both uORF1 and uORF2 (3) The uORF sequence is shown in the grey triangles, with the introduced variant in red text. **B** Luciferase activity of modelled uORF knock-out (start-loss) variants relative to wildtype. Bars represent mean with standard error of the mean. Knock-out of both *PKD1* 5′UTR uORFs increased luciferase activity significantly relative to wildtype (****p < 0.0001) (*p < 0.05).

To further characterise the role of the 5′UTR uORFs in *PKD1* regulation, we designed constructs that removed the start site for each uORF. We designed a construct that removed the start site of uORF1 alone, a construct that removed the start site of uORF2 alone and a third construct that removed the start site of both uORFs, all in the wildtype *PKD1* 5′UTR sequence (Fig. 4A). Luciferase activity of these modelled variants was then assessed (Fig. 4B). We identified that start-loss of uORF1 resulted in increased luciferase activity by 9% compared to wildtype (mean difference −0.09 *p* = 0.04, by Tukey HSD test). Start-loss of uORF2 alone resulted in increase in luciferase activity of 34% compared to wildtype (mean difference −0.34 *p* ≤ 0.0001 by Tukey HSD test). Loss of both start-sites resulted in the largest increase in luciferase activity, of 130% compared to wildtype (mean difference −1.31 *p* ≤ 0.0001, by Tukey HSD test).

## Discussion

We demonstrate that variants within the *PKD1* 5′UTR are a previously unrecognized cause of ADPKD and show an approach for assessing candidate variants identified in this region. We also demonstrate that single-base pair alterations of the wildtype 5′ UTR sequence leading to loss of the start codons of uORFs result in an increase in polycystin-1 expression in our cell model. Our results are significant for increasing diagnostic rates in ADPKD and in furthering our understanding of the role of the *PKD1* 5′UTR in polycystin-1 expression.

The majority of pathogenic variants causing typical ADPKD are located in coding regions of the *PKD1* gene and predicted to result in protein-truncation, with most other disease-causing variants being missense variants with predicted or proven loss-of-function effects [45]. After standard genomic testing, ~10% of individuals with ADPKD clinical features remain genetically undiagnosed [3]. Here, we report the first pathogenic 5′UTR variant in *PKD1* and add to the growing body of evidence which shows non-coding variation, such as 5′UTR variants, are an under-appreciated cause of monogenic disease [4]. By developing and validating a luciferase reporter gene assay to assess the *PKD1* 5′UTR, we found that the c.−69dupG variant, which was identified in a patient with a typical ADPKD phenotype, caused a significant reduction in the translational efficiency of the main ORF. The almost complete reduction in translation is consistent with this variant causing loss-of-function and haploinsufficiency of *PKD1*, which is consistent with the phenotype seen in the patient.

Rescue of the pathogenic effect of the c.−69dupG variant was demonstrated by introducing a stop codon to resume in frame translation termination at 58 (−59C>A, Rescue 1) and 7 (c.−8C>A, Rescue 2) nucleotides upstream of the mAUG. Whilst c.−59C>A rescued translation completely, c.−8C>A only partially rescued translation. It has been previously shown that translation from a main ORF can be dynamically regulated, with different uORFs having a greater or lesser ability to alter translation. Several factors, including the length of the uORF, sequence context, secondary structure, together with the distance of the uORF to both the 5′cap and CDS, and total number of uORFs have been proposed to influence the degree to which an uORF influences translation efficiency, and/or whether a uORF is bypassed [46, 47]. We considered multiple explanations for the incomplete rescue seen in the ‘Rescue 2′ model, which we now outline. If some baseline re-initiation is occurring naturally within this 5′UTR, the proximity between the introduced stop (‘Rescue 2’) and the main AUG may leave insufficient distance for the ribosome to reload and efficiently re-initiate translation [48]. Another consideration is whether the introduction of a variant in such proximity to the main AUG may have inadvertently altered the wildtype RNA structure surrounding the initiator AUG. In addition to the impact of the ‘Rescue 2’ variant on the main start site, it is also possible that this variant is impacting uORF2. The intended impact of the Rescue 2 variant may be compromised by the fact that the rescued uORF1 now terminates within uORF2. Though the stop codon and frame of uORF2 is unaltered by the Rescue 2 variant, the introduction of a new stop codon for uORF1 within uORF2 may be impacting the wildtype function of uORF2. Extension of uORF1 to within uORF2 may be impacting start initiation of uORF2.

Although our data demonstrates that the main pathogenic impact of the c.−69dupG 5′UTR variant is elongation of the uORF leading to decreased translation efficiency, without performing RNA studies, we cannot rule out additional effects the variant may have on pre-mRNA splicing and transcript stability. There is, however, no indication on in silico analysis that the c.−69dupG variant impacts splicing. Fresh urine samples required for RNA studies were unavailable.

Our data suggests that variants which disrupt uORF1, causing an overlapping ORF, are likely pathogenic. We did not identify any genomic variants found to disrupt uORF2, which is significantly closer to the translation start site of the main ORF. Nor did we identify common genomic variation in the gnomAD dataset that would support uORF2 being tolerant of variation. Given the complexities of uORF-mediated translation regulation, we reaffirm the view that in vitro or in vivo validation of any variant predicted to disrupt uORF1 and uORF2 or create a novel ORF is required to determine pathogenicity [16].

In our efforts to further characterise the regulation of *PKD1*, we identified that removing the start-site of either or both uORFs in the wildtype *PKD1* 5′UTR sequence results in increased polycystin-1 expression. Our results are consistent with a recent report by Chen et al. who also report that loss of the start site of either or both uORFs increases polycystin-1 expression [49]. Our data reiterates the findings in this publication by making different alterations to the start site. Given that haploinsufficiency is considered the basis of pathogenesis in ADPKD, avenues to increase expression of a wild-type allele provide an opportunity for treatment. Antisense oligonucleotides against uORFs have been used in other conditions to increase gene expression. Chen et al. trialled a single ASO combination targeting the uORF start sites, that resulted in reduction in polycystin-1 expression [49]. Studies utilising ASOs to manipulate *GATA4* expression in cardiac hypertrophy suggest that ASOs act to enhance or reduce protein expression through their impact on RNA structure rather than specific interaction with the uORF start-site, suggesting that further experimentation to identify an appropriate ASO sequence combination to manipulate polycystin-1 expression could be successful [24]. Our finding that single-base pair alterations in the 5′UTR sequence can increase expression could also mean that techniques such as RNA editing could be utilised to alter the mRNA 5′UTR sequence to increase PC1 expression of a wild-type allele [50]. In addition to potential long-term therapeutic potential, our finding open an avenue to further investigate ADPKD cyst pathogenesis by providing a means to increase cellular expression of polycystin-1 by harnessing the cell’s existing machinery.

Interrogation of undiagnosed ADPKD cohorts did not identify any additional disease-causing *PKD1* 5′UTR variants. We limited our analysis to the Genomics England rare disease cohorts and the Garvan cohorts as these are the only WGS-based undiagnosed ADPKD cohorts we are aware of, noting that exome and capture-based methods have poor coverage of the 5′UTR region [19]. The challenging nature of genomic analysis of the *PKD1* gene, including the GC-rich 5′UTR region, coupled with the fact that routine diagnostic testing does not typically assess non-coding regions, means that unascertained cases are a distinct possibility. Current variant interpretation guidelines typically focus on coding variants. Fewer guidelines exist for interpreting non-coding variants, with those that do more focussed on variants impacting splicing rather than those in regulatory regions [4]. Our data supports emerging data from other rare diseases that variants in the 5′UTR are a currently overlooked cause of disease that could be addressed through diagnostic testing. Systematic analysis of the 5′UTR as part of standard diagnostic genetic approaches would improve knowledge of the degree of variation in the region. The luciferase translation assay we describe is easily replicable and offers an avenue for in vitro assessment of uncertain variants in the region.

Improving genetic diagnostic rates has clear benefits for affected individuals and their families [33]. For RBW402, a genetic diagnosis allows screening of unaffected family members (and early institution of blood pressure surveillance if positive), provides information for reproductive decision making and can provide prognostic information [51]. A limitation of our analysis in RBW402’s family is that his parents were unavailable for testing to confirm that this variant is de novo—both parents had normal kidney ultrasounds at an older age, ruling out an ADPKD phenotype based on Pei imaging diagnostic criteria [52, 53].

Our findings reiterate the importance of considering non-coding regions in rare disease genomic analyses for the genetically undiagnosed and particularly assessing the *PKD1* 5′UTR for pathogenic variation in individuals with ADPKD. We demonstrate the role that the 5′UTR uORFs have in the regulation of PKD1 expression and show a method to increase wild-type expression. Experimental manipulation of the *PKD1* 5′UTR provides a realistic avenue to increase wild-type polycystin-1 expression and treat this haplo-insufficient disease.

## Supplementary information

Combined Supplementary Material

Supplementary Table 5

## Data Availability

The individual genomic datasets from the Garvan and KidGen cohorts generated and analysed during the current study are available from the corresponding author on reasonable request. Permission can be sought to access data from the Genomics England cohort through the Genomics England Research Environment. The public datasets analysed during the current study are available in the gnomAD repository, https://gnomad.broadinstitute.org. The variants identified in this study have been submitted to Clinvar (SUB15496204).
