# Supplementary material for: PKD1 5’UTR variants are a rare cause of disease in ADPKD and suggest a new focus for therapeutic development
Source: Eur J Hum Genet. 2025 Sep 26;34(1):61–9. doi: 10.1038/s41431-025-01949-z (PMC12816572; doi:10.1038/s41431-025-01949-z)
Supplement: Supplementary file 1 — Combined Supplementary Material [file 41431_2025_1949_MOESM1_ESM.pdf]

SUPPLEMENTARY DATA

SUPPLEMENTAL METHODS

Short read genome sequencing

Short-read genome sequencing was performed using DNA extracted from peripheral blood samples. Genome sequencing was performed on the HiSeqX sequencing system (Illumina Inc., California, CA, USA) after either PCR-based library preparation (Illumina HiSeq X TruSeq Nano DNA HT Sample Prep Kit) or PCR-free library preparation (KAPA Hyper PCR-free kit, Roche). The sequencing was performed within an ISO17025-accredited laboratory at the Kinghorn Centre for Clinical Genomics within the Garvan Institute.

All samples were processed via a custom bioinformatics pipeline based on GATK best practice, which was optimised for the identification of germline variants utilising the variant analysis platforms Seave or Seqr[1-3]. Where analysis was performed utilising Seave, reads were aligned to the hg37 reference sequence. Sequence variants were filtered using Seave. CNV and structural variant analysis was performed using ClinSV[4]. Splicing analysis was performed using Introme[5].

For instances where Seqr was utilised as the variant analysis platform, WGS data processing was performed by the Centre for Population Genomics following the DRAGEN GATK best practices pipeline. Reads were aligned to the hg38 reference genome using Dragmap (v1.3.0). Cohort-wide joint calling of single nucleotide variants (SNVs) and small insertion/deletion (indel) variants was performed using GATK HaplotypeCaller (v4.2.6.1) with "--dragen-mode" enabled. Variants were annotated using VEP 110, and loaded into the web-based variant filtration platform, Seqr. Sample sex and relatedness quality checks were

performed using Somalier (v0.2.15)[6] [4]. Structural variant (SV) calling from short-read whole-genome sequencing (WGS) data was performed using GATK-SV[7].

Long read sequencing

Where whole genome sequencing resulted in poor coverage of the *PKD1* 5'UTR, long-read sequencing was performed. High molecular weight DNA was sheared to ~20 kb fragment size using Covaris G-tubes. Sequencing libraries were prepared from ~1.5 to 5 µg of sheared DNA using native library prep kits (SQK-LSK110) and sequenced for 72 h on a PromethION (FLO-PRO002, R9.4.1) flow cell. Raw ONT sequencing data was converted to BLOW5 format with slow5tools (v0.3.0)[8], then base-called using Guppy (4.0.11 or later). Resulting FASTQ files were aligned to the hg38 reference genome using minimap2 (v2.14-r883)[9], and Longshot (v0.4.1)[10] was used to identify and phase variants within the *PKD1* locus. Long-read sequencing was performed within the Garvan Institute.

Sanger sequencing

Sanger sequencing (with prior LR-PCR amplification if within the PKD1-pseudogene homologous region) was performed to confirm all single nucleotide and short indel variants identified on genome and/or long read sequencing.

SUPPLEMENTAL RESULTS

Section 1: GnomAD PKD1 5'UTR and uORF variation

We assessed all gnomAD v4.0 variants of an allele frequency of <6e-05 to determine whether any potential AUG-creating or uORF-disrupting variants occurred in the database

(Supplemental Table 4). Variation of any allele frequency across both uORF1 and uORF2 was also assessed (Supplemental Table 5). No stop loss variants of ORF1 or ORF2 of any allele frequency or rare AUG-creating variants that were predicted to cause read through of the main ORF were identified AUG start loss variants were observed for both ORF1 (c.-86T>A and c.-87A>G) and ORF2 (c.-19T>C and c.-20A>T). Missense variation was observed in both uORFs. A single AUG-creating variant was identified, chr16:2135691-G-A (c.-2C>T) and classified as uncertain significance, as it is predicted to cause a 1 amino acid N-terminal extension. It also occurs within the Kozak consensus sequence of the main ORF, altering the –2 position (C**TAACG**_ATG_C> C**TAA*T*G**_ATG_C); weaking of the translational efficiency of the main ORF cannot be excluded.

References

1. Gayevskiy V *et al*: Seave: a comprehensive web platform for storing and interrogating human genomic variation. *Bioinformatics* 2019; **35:** 122-125.

2. Pais LS *et al*: seqr: A web-based analysis and collaboration tool for rare disease genomics. *Hum Mutat* 2022; **43:** 698-707.

3. Hort Y *et al*: Atypical splicing variants in PKD1 explain most undiagnosed typical familial ADPKD. *npj Genomic Medicine* 2023; **8:** 16.

4. Minoche AE *et al*: ClinSV: clinical grade structural and copy number variant detection from whole genome sequencing data. *Genome Medicine* 2021; **13:** 32.

5. Sullivan PJ *et al*: Introme accurately predicts the impact of coding and noncoding variants on gene splicing, with clinical applications. *Genome Biology* 2023; **24:** 118.

6. Pedersen BS *et al*: Somalier: rapid relatedness estimation for cancer and germline studies using efficient genome sketches. *Genome Med* 2020; **12:** 62.

7. Collins RL *et al*: A structural variation reference for medical and population genetics. *Nature* 2020; **581:** 444-451.

8. Gamaarachchi H *et al*: Fast nanopore sequencing data analysis with SLOW5. *Nat Biotechnol* 2022; **40:** 1026-1029.

9. Li H: Minimap2: pairwise alignment for nucleotide sequences. *Bioinformatics* 2018; **34:** 3094-3100.

10. Edge P *et al*: Longshot enables accurate variant calling in diploid genomes from single-molecule long read sequencing. *Nat Commun* 2019; **10:** 4660.

Supplemental Figure 1: IGV Capture of variant identified in RBW402.

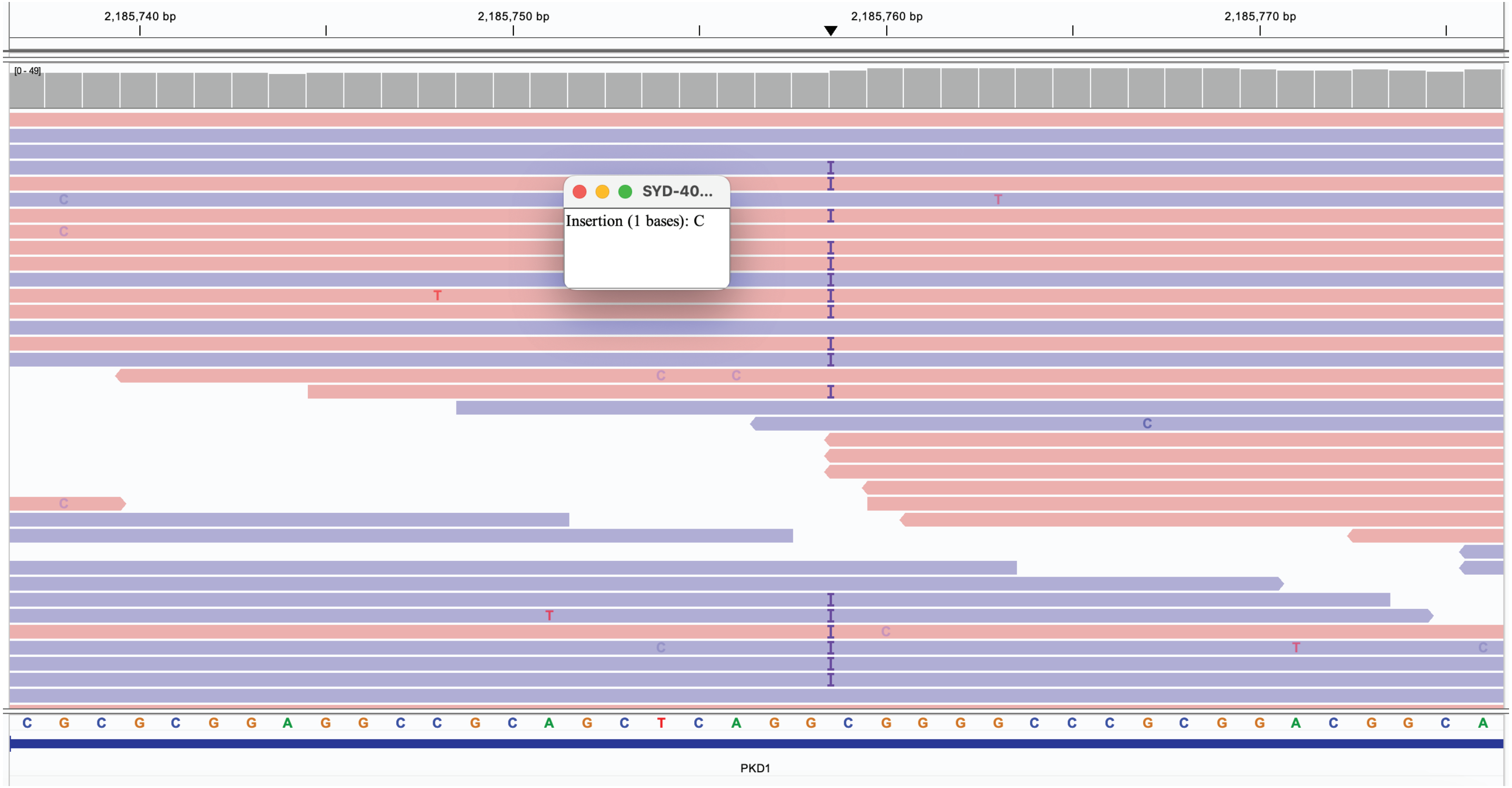

Supplemental Figure 1: IGV Capture of variant identified in RBW402.
NM_001009944.3:c.-69dupG, NC_000016.9:g.2185757_2185758insC (Feb.2009: hg19, GRCh37)
or NC_000016.10:g.2135757_2135758insC (Dec.2013: hg38, GRCh38). Aligned in this image to GRCh37.

Supplemental Table 1: 5'UTR Wildtype and variant constructs

Name	Variant genomic coordinates (GRCh38)	Unique patient identifier	Variant interpretation	Construct sequence (5'UTR)	Predicted impact
**Wildtype PKD1 5'UTR sequence**	N/A	N/A	Wildtype PKD1 5'UTR sequence	GCACTGCAGCGCCAGCGTCCGAGCGGGCGGCCGAGCTCCC<br>GGAGCGGCCTGGCCCCGAGCCCCGAGCGGGCGTCGCTCA<br>GCAGCAGGTCGCGGCCGCAGCCCCATCCAGCCCCGCGCC<br>CGCCATGCCGTCCGCGGGCCCCGCCTGAGCTGCGGCCTCC<br>GCGCGCGGGCGGGCCTGGGGACGGCGGGGCCATGCGCGC<br>GCTGCCCTAACG	uORF1: MPSAGPA[stop], uORF2 MRALP[stop]
**c.-69dupG**	chr16:2135757G>GC	RBW402	Predicted as likely uORF-disrupting based on: (1) Insertion (*underlined/italicised*) within uORF1 causing a frameshift and stop loss (uORF1 stop bolded), space signifies reading frame: GCC *ATG* CCG TCC GCG GGC CCC G*G* C C**TG A** (2) Absent from gnomAD v4.0.0	GCACTGCAGCGCCAGCGTCCGAGCGGGCGGCCGAGCTCCCGGAG<br>CGGCCTGGCCCCGAGCCCCGAGCGGGCGTCGCTCAGCAGCAGGTC<br>GCGGCCGCAGCCCCATCCAGCCCCGCGCCCGCCATGCCGTCCGC<br>GGGCCCCGGCCTGAGCTGCGGCCTCCGCGCGCGGGCGGGCCTGG<br>GGACGGCGGGGCCATGCGCGCGCTGCCCTAACG	oORF for *PKD1* (underlined text signifies portion overlapping with main ORF, 112 amino acid readthrough) MPSAGPGLSCGLRARAGLGTAGPCARCPNDAARRARPPGAGPGPGPVAR GAGGGPRARLRALRAPLPLRPSARRRLPRQLLGPRAADARSRAAHPRGRH SARRLPQPAPGAGRWAPGEPL GAGRAGYKQQQDFYVRRRNIC[stop]
**Rescue c.-59C>A**	chr16:2135757G>GC, in-phase with chr16:g.2135748G>T		Downstream stop gain to rescue c.-69dup. In-phase nucleotide substitution c.-59C>A introducing an in-frame stop codon.	GCACTGCAGCGCCAGCGTCCGAGCGGGCGGCCGAGCTCCCGGAG<br>CGGCCTGGCCCCGAGCCCCGAGCGGGCGTCGCTCAGCAGCAGGTC<br>GCGGCCGCAGCCCCATCCAGCCCCGCGCCCGCCATGCCGTCCGC<br>GGGCCCCGGCCTGAGCTGAGGCCTCCGCGCGCGGGCGGGCCTGG<br>GGACGGCGGGGCCATGCGCGCGCTGCCCTAACG	oORF rescue: MPSAGPGLS[stop]
**Rescue c.-8C>A**	chr16:g.2135697G>T		Downstream stop gain to rescue c.-69dup. In-phase nucleotide subtitution GRCh38(chr16):g.2135697G>T (c.-8C>A) introducing an in-frame stop codon close to the main ORF AUG.	GCACTGCAGCGCCAGCGTCCGAGCGGGCGGCCGAGCTCCCGGAG<br>CGGCCTGGCCCCGAGCCCCGAGCGGGCGTCGCTCAGCAGCAGGTC<br>GCGGCCGCAGCCCCATCCAGCCCCGCGCCCGCCATGCCGTCCGC<br>GGGCCCCGGCCTGAGCTGCGGCCTCCGCGCGCGGGCGGGCCTGG<br>GGACGGCGGGGCCATGCGCGCGCTGACCTAACG	oORF rescue 2: MPSAGPGLSCGLRARAGLGTAGPCAR[stop]
**Benign c.-52C>T**	chr16:g.2135741G>A c.-52C>T	N/A	Predicted as benign based on: (1) high frequency in population databases (gnomAD v4.0.0 total allele frequency 0.002536, African/African American allele frequency 0.03635) (2) not predicted to disrupt uORF and/or introduce AUG (3) not predicted to impact splicing (SpliceAI, Pangolin 0.0).	GCACTGCAGCGCCAGCGTCCGAGCGGGCGGCCGAGCTCCCGGAG<br>CGGCCTGGCCCCGAGCCCCGAGCGGGCGTCGCTCAGCAGCAGGTC<br>GCGGCCGCAGCCCCATCCAGCCCCGCGCCCGCCATGCCGTCCGC<br>GGGCCCCGCCTGAGCTGCGGCCTCTGCGCGCGGGCGGGCCTGGG<br>GACGGCGGGGCCATGCGCGCGCTGCCCTAACG	Benign 1: uORF1 undisrupted MPSAGPA[stop], uORF#2 undisrupted MRALP[stop]
**NHS1 c.-209G>A**	chr16:g.2135898C>T	NHS1	In silico analysis alone predicted this variant as likely benign based on: (1) not predicted to disrupt uORF and/or introduce AUG (2) not predicted to impact splicing (SpliceAI, Pangolin 0.0). However, this variant is observed in gnomAD v4.0.0 at a low frequency (East Asian subpopulation 0.0001892) not meeting benign supporting based on allele frequency alone. This proband was also noted to have a PKD1 missense variant of uncertain significance p.Arg2186Pro (ENST00000262304.4: c.6557G>C, ClinVar variation ID: 976821) that has been observed in one other unrelated proband with ADPKD. As such, initial variant curation classed this variant as of uncertain significance.	ACACTGCAGCGCCAGCGTCCGAGCGGGCGGCCGAGCTCCCGGAGC<br>GGCCTGGCCCCGAGCCCCGAGCGGGCGTCGCTCAGCAGCAGGTCG<br>CGGCCGCAGCCCCATCCAGCCCCGCGCCCGCCATGCCGTCCGCG<br>GGCCCCGCCTGAGCTGCGGCCTCCGCGCGCGGGCGGGCCTGGGG<br>ACGGCGGGGCCATGCGCGCGCTGCCCTAACG	Likely benign/uncertain: uORF1 undisrupted MPSAGPA[stop], uORF#2 undisrupted MRALP[stop]

Supplemental Table 2: Rare PKD1 and PKD2 variants detected in RBW402

Variant (GRCh37)	Variant (GRCh38)	HGVS.c	Type	Impact	VAF	gnomAD v4.0.0 Allele Frequency	SpliceAI predicted impact	5'UTR predicted impact
4-88969889-TACA-T	4-88048737-TACA-T	ENST00000237596.2:c.1548+1871_ 1548+1873delACA	Deletion	intron_variant	0.56 (19/34)	0.00009857	<0.05	N/A
16-2154926-G-A	16-2104925-G-A	ENST00000262304.4:c.8017-283C>T	SNP	intron_variant	0.75 (3/4)	0.00008652	<0.05	N/A
16-2185758-G-GC	16-2135757-G-GC	ENST00000262304.4:c.-69dupG	Insertion	5_prime_UTR_variant	0.67 (20/30)	Absent	<0.05	Out-of-frame overlapping reading frame predicted

Supplemental Table 3: Rare *PKD1* 5'UTR variants listed in ClinVar and/or PKD database (accessed 31/01/2024)

**Variant (GRCh38)**	**HGVS.c (ENST00000262304)**	**gnomAD v4.0.0 allele frequency (total)**	**Rare disease cohort/database**	**5'UTR predicted impact***	**uORF impact**	**ClinVar ID**	**ClinVar classification**
16-2135741-G-A	-52C>T	0.002536	PKD database	Likely benign	None	997323	Benign
16-2135750-A-G	-61T>C	0.0000363	PKD database	Likely benign	None	N/A	
16-2135751-G-A	-62C>T	0.0003634	ClinVar	Likely benign	None	997348	Likely benign
16-2135756-G-A	-67C>T	0.001044	PKD database	Likely benign	uORF1 synonymous variant (Ala>Ala)	433937	Likely benign
16-2135765-C-G	-76G>C	0.0000546	PKD database	Likely benign	None	N/A	
16-2135797-G-A	-108C>T	0.008369	PKD database	Likely benign	None	N/A	
16-2135798-G-C	-109C>G	Absent	ClinVar	Likely benign	None	997208	Likely benign
16-2135798-G-A	-109C>T	0.0007739	ClinVar	Likely benign	None	997324	Benign
16-2135806-C-A	-117G>T	Absent	PKD database	Likely benign	None	N/A	
16-2135876-C-T	-187G>A	0.00001788	ClinVar	Likely benign	None	997127	Likely benign
16-2135884-C-T	-195G>A	0.0007583	ClinVar	Likely benign	None	433936	Likely benign
16-2135896-T-C	-207A>G	0.0000207	ClinVar	Likely benign	None	997237	Likely benign

*5'UTR variants were assessed for AUG-creating or uORF disrupting impacts. Variants not predicted to introduce a novel AUG or disrupt uORF1 or uORF2 were classified as likely benign

Supplemental Table 4: All reported variation in Gnomad v4.1 within uORF1 (chr16(GRCh38):2,135,753-2,135,776) and uORF2 chr16(GRCh38):2,135,692-2,135,709

uORF	gnomAD ID	Chromosome	Position	Reference	Alternate	HGVS Consequence	Allele Count	Allele Number	Allele Frequency	Homozygote Count	cadd	spliceai_ds_max	pangolin_largest_ds	Codon change	uORF predicted impact
uORF1	16-2135756-G-A	16	2135756	G	A	c.-67C>T	1004	961396	0.00104432	16	11.5	0	0	gcc>gcT	Gly>Gly (synonymous)
uORF1	16-2135757-G-T	16	2135757	G	T	c.-68C>A	2	959228	2.09E-06	0	13.1	0	0	gcc>gAc	Gly>Asp
uORF1	16-2135757-G-A	16	2135757	G	A	c.-68C>T	4	959228	4.17E-06	0	13.6	0	0	gcc>gTc	Gly>Val
uORF1	16-2135761-G-A	16	2135761	G	A	c.-72C>T	1	958520	1.04E-06	0	5.31	0	0	ccc>Tcc	Pro>Ser
uORF1	16-2135762-G-C	16	2135762	G	C	c.-73C>G	3	953874	3.15E-06	0	8.6	0	0	ggc>ggg	Gly>Gly (synonymous)
uORF1	16-2135762-G-A	16	2135762	G	A	c.-73C>T	31	953874	3.25E-05	0	8.89	0	0	ggc>ggt	Gly>Gly (synonymous)
uORF1	16-2135763-C-A	16	2135763	C	A	c.-74G>T	1	954610	1.05E-06	0	5.55	0	0	ggc>gTc	Gly>Val
uORF1	16-2135763-C-T	16	2135763	C	T	c.-74G>A	4	954502	4.19E-06	0	6.2	0	0	ggc>gAc	Gly>Asp
uORF1	16-2135764-C-A	16	2135764	C	A	c.-75G>T	1	953228	1.05E-06	0	9.19	0	0	ggc>Tgc	Gly>Cys
uORF1	16-2135765-C-G	16	2135765	C	G	c.-76G>C	52	952370	5.46E-05	0	3.03	0	0	gcg>gcC	Ala>Ala (synonymous)
uORF1	16-2135766-G-T	16	2135766	G	T	c.-77C>A	20	950020	2.11E-05	1	6.88	0	0	gcg>gAg	Ala>Asp
uORF1	16-2135768-G-A	16	2135768	G	A	c.-79C>T	3	943124	3.18E-06	0	9.37	0	0	tcc>tcT	Ser>Ser (synonymous)
uORF1	16-2135772-G-C	16	2135772	G	C	c.-83C>G	5	927380	5.39E-06	0	6.6	0.02	0.01	ccg>cGc	Pro>Arg
uORF1	16-2135772-G-A	16	2135772	G	A	c.-83C>T	6	927274	6.47E-06	0	6.87	0.01	-0.01	ccg>cTg	Pro>Leu
uORF1	16-2135775-A-T	16	2135775	A	T	c.-86T>A	2	910830	2.20E-06	0	14.3	0	0	atg>aAg	Start loss
uORF1	16-2135776-T-C	16	2135776	T	C	c.-87A>G	12	911150	1.32E-05	0	14.7	0	0	atg>Ctg	Start loss (CUG initiation cannot be excluded)
uORF2	16-2135695-G-A	16	2135695	G	A	c.-6C>T	33	863378	3.82E-05	0	16.6	0	0	ccc>ccT	Pro>Pro (synonymous)
uORF2	16-2135697-G-A	16	2135697	G	A	c.-8C>T	7	872754	8.02E-06	0	16.8	0	0	ccc>Tcc	Pro>Ser
uORF2	16-2135698-C-A	16	2135698	C	A	c.-9G>T	3	877362	3.42E-06	0	16.3	0	0	ctg>ctT	Leu>Leu (synonymous)
uORF2	16-2135698-C-T	16	2135698	C	T	c.-9G>A	4	877364	4.56E-06	0	16.7	0	0	ctg>ctA	Leu>Leu (synonymous)
uORF2	16-2135700-G-C	16	2135700	G	C	c.-11C>G	13	885084	1.47E-05	0	16.1	0	0	ctg>Gtg	Leu>Val
uORF2	16-2135701-C-G	16	2135701	C	G	c.-12G>C	2	892736	2.24E-06	0	15.5	0	0	gcg>gcC	Ala>Ala (synonymous)
uORF2	16-2135702-G-C	16	2135702	G	C	c.-13C>G	1	894930	1.12E-06	0	16.1	0	0	gcg>gGg	Ala>Gly
uORF2	16-2135703-C-T	16	2135703	C	T	c.-14G>A	1	897842	1.11E-06	0	15.9	0	0	gcg>Acg	Ala>Thr
uORF2	16-2135703-C-G	16	2135703	C	G	c.-14G>C	1	897946	1.11E-06	0	15.6	0	0	gcg>Ccg	Ala>Pro
uORF2	16-2135708-A-G	16	2135708	A	G	c.-19T>C	2	915060	2.19E-06	0	17.9	0	0	atg>aCg	Start loss
uORF2	16-2135709-T-A	16	2135709	T	A	c.-20A>T	2	911806	2.19E-06	0	18.3	0	0	atg>Ttg	Start loss
